# The joy of eating: how eating experiences enhance the well-being of older adults

**DOI:** 10.3389/fpubh.2024.1438964

**Published:** 2024-09-09

**Authors:** Xinmin Wang, Jianwu Qi, Kai Zhang, Huiji Xie, Xingnan Wu

**Affiliations:** ^1^Colleges of Resource and Environmental Engineering, Tianshui Normal University, Tianshui, China; ^2^Department of Tourist Management, South China University of Technology, Guangzhou, China; ^3^Colleges of Architecture, Xi’an University of Architecture and Technology, Xi’an, China

**Keywords:** eating experience, CAPS, nostalgia, place attachment, older adults’ well-being

## Abstract

**Introduction:**

The active aging strategy has as its policy implications the health, security, and participation of older people. The joy of eating is the main goal of establishing community-based service facilities for older people, as well as a source of health and well-being and a sense of meaning in the lives of older people.

**Methods:**

Based on the theory of human-environment relations and cognitive-emotional personality systems (CAPS), the study constructed a structural equation model of the eating experience, nostalgia, place attachment, and the well-being of the older adults in the community canteens as an interactive situation, and explored the relationship between the eating experience and the well-being of older people in the community canteens through the partial least squares structural equation modeling (PLS-SEM).

**Results:**

The results of the study show that the older adults’ eating experience has a significant positive effect on their well-being, and “eating” can make older adults feel happy. Older adults’ eating experience has a significant effect on nostalgia, place attachment, and well-being, but nostalgia does not have a significant effect on older adults’ well-being, and place attachment in the community canteens can enhance older adults’ well-being. Meanwhile, the study further confirmed that place attachment plays a mediating role in the effect of eating experience on older adults’ well-being.

**Discussion:**

The findings of the study promote the development of the fields of healthy eating, quality of life assessment, and dietary memory management for older people to a certain extent and provide an important reference for promoting the balanced layout and effective spatial design of community service facilities for older people.

## 1 Introduction

Food changes mark epochal social changes in a wide range of contexts, and as an important dimension of temporal, spatial, and social examination, food can both characterize the past and examine the present in terms of the past (1). Since the end of the 20th century, research on the Chinese diet has entered a phase of rapid development. Different scholars have focused extensively on the study of dietary customs and rituals, regional changes in daily dietary life, dietary and cultural characteristics and techniques, and the relationship between people and their diets in various historical periods (2–4). In the 21st century, with the strengthening of the trend of economic and trade globalization and the increase of cultural integration and exchanges between different countries and regions, the focus of traditional Chinese dietary research in cross-cultural and interdisciplinary contexts has been on food health and safety, dietary life and behavior, Chinese and Western dietary communication, and dietary symbols and symbolic meanings (5–7), most of the studies have shifted the perspective to the eating habits and behaviors of individuals in their daily lives, focusing on the construction of food meanings, rights and identities. Food is endowed with multiple values and characteristics in different political, cultural, and economic contexts, and exploring the intrinsic relationship between eating experience and well-being is crucial for the inheritance of regional food culture and the enhancement of cultural identity.

Not only does food produce physiological experiences such as lethargy, hunger, illness, and discomfort, but it also interferes with psychological experiences, both of which can influence a person’s sense of well-being (8, 9). In China’s current policy context, dietary health and eating safety of the older adults play an essential role in promoting the implementation of the active aging strategy. Chinese President Xi Jinping has repeatedly mentioned that “the key to measuring the well-being of a society lies in the level of the well-being perceived by the older adults.” Between 1964 and 2020, China’s share of the world’s older adult population aged 65 and over grew from 14.8 to 25.6%, representing almost one Chinese out of every four older adults worldwide. The large population base and rapid growth rate have enabled China to enter a profoundly aging society early. However, the number of empty nesters and older adults living alone has reached 118 million, leading to a weakening of the family’s function of providing for the older adults and an increase in the risk of illness among older adults. A survey on the mental health of the older adults in China conducted by the Institute of Psychology of the Chinese Academy of Sciences found that about 72.7% of the older adults had an excellent subjective sense of well-being and were happy and satisfied with their lives. In comparison, 9.7% felt sad and depressed about their lives, and the well-being of the older adults in China as a whole was at a medium to high level. For older adults, good eating experience and dietary habits can help to enhance their life health and life satisfaction and is an important path to heal older adults’ inner trauma and gain a sense of well-being (10–13). Due to the extreme physical and emotional vulnerability of older adults, factors such as food variety, food quality and chewing ability, frequency of food intake, and dietary environment can impact older adults’ well-being (14). Therefore, it is essential to explore how eating experience affects the well-being of older adults as China is about to enter a stage of super-aging.

However, previous studies have somewhat neglected the subjectivity of older adults in food consumption, overemphasizing the impact of food itself on older adults’ physical health and lacking an analysis of older adults’ emotional value of food. Meanwhile, despite the growing number of studies focusing on eating experience, food culture, and food safety, more needs to be explored regarding the relationship between eating experience, nostalgia, and place attachment and older adults’ well-being. In this context, the study integrated human-environment relations and cognitive-emotional personality systems theories, took the intrinsic relationships among eating experience, nostalgia, place attachment, and older adults’ well-being as a framework, and synthesized a structural equation model as a means of exploring the effects of eating experience on older adults’ well-being. Specifically, the relationship between the role of eating experience on nostalgia, place attachment, and older adults’ well-being was first examined, then the process of the influence of nostalgia and place attachment on older adults’ well-being was assessed, and finally, the mediating role of nostalgia and place attachment in the relationship between eating experience and older adults’ well-being was analyzed. Overall, the study established three main objectives: first, to explore older adults’ eating habits and emotional states in specific scenarios. The second is to explain the process of the influence of eating experience, nostalgia, and place attachment on the well-being of older adults. Third, to provide empirical evidence for forming healthy eating habits and enhancing life satisfaction among older adults. Since the study focuses on the process of the role between older adults’ eating experience in the community canteens and their emotional state, memorable experience, and life satisfaction, this study will provide certain innovative perspectives and theoretical references for the fields of older adults’ healthy eating, quality-of-life assessment, and the layout of community-based service facilities for older people.

## 2 Literature review and hypothesis

### 2.1 Food and eating experiences

The symbol of food as fuel is linked to the symbol of the body as a “machine” (15). Increased attention to physical experience, including the heterogeneous experience of food, will inevitably lead to a richer vocabulary of the body, the brain, and their relationship to each other, resulting in new ways of thinking about food. Body sense in the eating experience is a sensory habitus and cognitive pattern developed through repeated training over time, consciously or unconsciously, in everyday life (16). Once food enters a person’s body and is digested, it stimulates the body’s sensory organs, eventually translating into a physiological and psychological response to food. Long-term repetition of the same kind of eating behavior will train specific bodily sense items or patterns (categorization), and when the body encounters the same food stimulus again, the bodily sense items will be awakened in a specific context, which triggers the individual’s recollection of information about the food, the event, and the environment to produce or reinforce emotional identification (17, 18). The symbolic meaning and sensory value of food carry the individual’s experience and recollection of past historical events (5) and enhance/offsets the individual’s pervasive perceptions of collectively shared values through the positive or negative nostalgia brought about by the eating experience (19, 20); and at the same time, under the regional culture, the environment stimulates the individual’s identity, ethnicity, place, and cultural identity (21, 22). It has been shown that food-related nostalgia and place attachment are important representations of the eating experience (23–26).

Nostalgia, first introduced as a technical term in the 17th century, has long been recognized as a sentimental longing for personal past experiences (27). Before the end of the 20th century, nostalgia usually had a negative or negative connotation and was considered a manifestation of mental illness and depression (24, 28–30). Nostalgia is a complex emotional experience that is mostly seen as a reproduction of positive emotions (31), despite having a negative emotional component and is associated with hope, optimism, gratitude, warmth, and well-being (32, 33). Related studies have suggested that the development of nostalgia is closely related to food behavior, eating experience, and regional food culture (6, 34, 35). For different regions of multiethnic countries, influenced by language, history, and cultural differences, each region’s food culture and customs show different characteristics, leading to different nostalgia. For example, the ancestors of the inhabitants of the Macao region had close ties with Portugal and mainland China, and the food culture of the Macao region contains the main characteristics of Cantonese cuisine and absorbs the traditional flavors of Mediterranean cuisine. In a study on the eating experience in the Macau region, it was found that the Hong Kong night market, which focuses on food, can make people feel nostalgic. The food and atmosphere in the market can stimulate nostalgic feelings in tourists, which in turn affects their positive attitudes towards Macau’s food and the formation of their local identity with Macau (36). However, for the Tibetan region, Tibetan food has a special symbolic meaning in Tibetan cultural beliefs. In the Tibetan dietary structure and habits, tsampa, ghee tea, and sweet tea are highly representative traditional delicacies that satisfy the nutrients needed for life and, at the same time, express the symbolic significance of specific ethnic boundaries in some important occasions, festivals and ceremonies (37). In summary, food can directly trigger nostalgia through specific types of consumption behaviors, i.e., food nostalgia (38); when people consume nostalgic foods, they will experience more intense positive emotions, which typically include childhood, yearning, vicariousness, thoughts, special occasions, and rediscovery. From this, the following hypothesis is proposed:

H1: Eating experiences have a positive effect on nostalgia in older adults.

The impact of eating experiences on place attachment is closely related to regional food and cultural identities associated with food (39). On the one hand, regional foods and cuisines reflect the regional environment and cultural characteristics and activate the group’s inner interest in regional foods and food cultural identity (40, 41). In the process of participating in regional food activities and events and tasting regional food, people stimulate the group’s curiosity about regional eating experiences through the fulfillment of self-body functions and sensory enjoyment; and deepen their sense of identity and belonging to the place while participating in the construction of the regional food culture system (42, 43). For example, in addition to the influence of natural conditions, production technology, and socially prescribed factors, the learning, imitation, and assimilation of Japanese food culture from outside food circles were also important factors influencing the changes in Japanese food culture after World War II (44). At the same time, when Japanese people accept foreign food cultures, they will improve them according to the cultural habits that have been passed down from generation to generation in Japan, which is an important reason why Japan can quickly become the world’s gastronomic nation (14). In the Japanese concept of national identity, the national flag and “Washoku” are symbolic systems that mark differences in identity. In Southeast Asia, Indian eating habits are closely related to symbolism, cultural characteristics, and different expressions of social identity. Indians have a rich vegetarian tradition and believe in vegetarianism because, in the Indian cognitive world, vegetarianism represents purity and is ethical; also, vegetarian foods are rich in dietary fiber, vitamins, and minerals, which are beneficial for the prevention of chronic diseases (45).

On the other hand, place attachment is inseparable from food nostalgia and “place” is an indispensable element of nostalgia, which is not nostalgia for a specific place, but a way of life and existence (46). This way of life reflects the dichotomy between “space” and “place” in modern society, where space symbolizes freedom and implies a threat, while place implies security and creates meaning for life (47). This is reflected both in the social construction and place identity of nostalgic restaurants and consumption spaces (48), and in the sense of security and belonging that is formed in the relational interactions between human and external environments, such as their homes, neighborhoods, and cities (49). For example, Italy, Spain, Greece, and Morocco share a common Mediterranean regional history and food culture identity. The Mediterranean diet is both a way of life and a cultural fusion that consists mainly of sharing food and eating together (50). By sharing food, tastes, and traditions, people in the Mediterranean region use the Mediterranean diet as a critical element of regional common belonging and identity; at the same time, shared meals are an essential basis for food culture identity, in line with the need for collective continuity in the Mediterranean region. Italy is an important birthplace of Mediterranean cuisine, and pizza and pasta are highly symbolic in constructing Italian national identity. The variety and healthiness of Italian cuisine, the comfort and pleasure of Italian restaurants, and the romantic concept of the sweet life of Italians promote the spread and exchange of Italian food culture in the global region (51). It can be seen that the eating experience establishes a social connection with place through consumption behavior. When food is the main product, and a positive consumption experience is gained, people’s experiences and memories are reinforced, and their sense of identity and belonging to the regional culture increases. Based on this, the study proposes the hypothesis:

H2: Eating experiences have a positive impact on place attachment in older adults.

### 2.2 Factors affecting the well-being of older adults

Older adults’ well-being is an optimal psychological functioning and experience possessed by the older adult population (9), including older adults’ emotional responses, domain satisfaction, and global judgment of life satisfaction (33). Established studies have suggested that the well-being of older adults is influenced by a combination of external objective and internal subjective factors (52). External objective factors include family status, life events, health status, marital quality, cultural differences, and social support. Internal subjective factors include personality traits, intelligence level, and cognitive patterns. From the current trend of research on the factors influencing the well-being of older adults, the fulfillment of the basic needs of older adults is positively related to well-being (53). In this process, food consumption intervenes in changes in well-being by affecting the physical and mental health of older adults. Food can modulate consumers’ health through sensory and perceptual processes in the body and further influence individuals’ physical and psychological pleasure and emotions (54, 55). In most cases, consumers cite food and health, pleasure, and well-being as important antecedents of food consumption (56). For example, Vega-Zamora et al. argue that food consumption consists of both physiological and psychological experiences and that consumers’ expectations of food quality and physical well-being are more likely to influence how the consumption experience feels (57). In addition to the influence of the basic needs of older people, family income and expenditure, living environment, and social support of older people are also important factors that intervene in the well-being of older adults (52). For example, family recipes are associated with financial income, and good or bad financial conditions determine the quality of older adults’ diets and modulate their perceived level of well-being. In addition, other life events, such as bereavement, widowhood, unemployment, and disability versus living alone, have also been shown to be associated with chronically lower levels of well-being (33).

Older adults’ well-being is not only reflected in older people’s fulfillment of the basic needs of daily life but also the self-perception of positive and negative emotions at the level of psychological needs (9, 24). Rational experiences and subjective emotions shape consumers’ food choices, and the satisfaction and fulfillment associated with healthy food choices contribute to psychological well-being (58). Health concerns have been used as a moderating variable to explore the effects of food consumption on well-being (59). Health-conscious consumers are more likely to experience a reduction in health-related worries and anxieties from food consumption, as well as an increase in satisfaction with food choices, and a significant increase in individual perceptions of food well-being. With the changes in physical functioning and social status of older adults, they face the dual dilemmas of declining physical health and fading social relationships and are more prone to negative emotions, such as depression, loneliness, aging, and low self-esteem. Current research has found that food can awaken happy or sad emotions and enhance people’s sense of well-being and emotional experience (55, 60, 61). There is a strong relationship between different types of food preferences, consumption, and negative and positive emotions (62); positive emotions increase a person’s willingness to try or indulge in food, while negative emotions decrease a person’s willingness to try a healthy food. Therefore, the hypothesis is proposed:

H3: Eating experiences have a positive impact on the development of well-being in older adults.

Nostalgia not only increases an individual’s sense of meaning in life through social connectedness, personal development, and self-esteem (63); it also promotes physical and mental well-being and strengthens an individual’s intrinsic motivation and work effort (64). For older people, the association between nostalgia and well-being is directly reflected in aspects such as famine experiences and psychological trauma (65), with famine experiences influencing middle-aged and older adults’ food consumption and intrinsic emotions by raising the psychological price and expectations of food. In terms of mental health, food-induced nostalgia can re-heal older adults’ psychological trauma and create a fictionalized childhood. Gratitude, meaning of life, and authenticity are mostly used as mediators to explore the relationship between nostalgia and well-being (32, 64), while the relationship between nostalgia and well-being among older adults has received less attention. Nostalgia for food in older adults awakens memories of past experiences, stimulates psychological experiences in the life course, and correlates this nostalgia and psychological experiences with real life as a way to gain a sense of meaning and well-being about life. As a result, the following hypotheses are proposed:

H4: Nostalgia has a positive impact on the development of well-being in older adults.

The emotional valence of the cognitive subject in the eating experience is not only reflected in the physiological and psychological needs for food but also in the intrinsic interaction between the subjects of experience and the environment in which the food is coexists (34). Current research has confirmed that there is a close association between place attachment and well-being that older adult’s dependence and sense of belonging are conducive to maintaining and enhancing their sense of well-being and that place attachment positively affects well-being (66). Well-being, in turn, counteracts one’s cognitive experience of place and environment, providing individuals with a sense of belonging, purpose, and meaning in life (67, 68). Meanwhile, psychological factors such as life satisfaction and happiness are stronger predictors of place attachment (69). Based on the above discussion, the following hypotheses are proposed:

H5: Place attachment has a positive impact on the development of well-being in older adults.

### 2.3 Eating experience, nostalgia, place attachment, and older adults’ well-being

In traditional Chinese eating culture and consumption concepts, food not only has an external visual and sensible nature but also has an internal symbolic and metaphorical nature; in the framework of social interactions and cultural values centered on food, the act of eating has become a basic way of perceiving and grasping the world. However, as an important theme in the study of eating culture and well-being, eating experience and the well-being of older adults have seldom been explored in the same system. Current research suggests that the effect of eating experience on well-being is closely related to individuals’ subjectively perceived physical health, happiness, and emotional aspects (55, 56, 70). Differences in older adults’ physical experience and psychological perception of food and their eating behaviors are influenced by factors such as environment and food color in the space, which makes older adults’ sense of belonging and identity to the place biased and indirectly affects their affective experience and well-being perceptions of a specific space. Older adults’ eating experiences enhance their sense of belonging and identification with food and eating environments, and correlate with older adults’ past experiences under the influence of factors such as family relationships, income levels, physical conditions, and educational backgrounds to form emotional contrasts with current situations and people, which ultimately translates into judgments of well-being (71, 72). The above discussion suggests that eating experiences not only stimulate positive nostalgia in older adults (23, 28, 34), but also enhance older adults’ sense of security, identity, and belonging to a place, and strengthen their place attachment (24, 33, 73). Therefore, the following hypothesis is proposed:

H6: Eating experiences indirectly affect older adults’ well-being through the intermediation role of nostalgia.

H7: Eating experiences indirectly affect older adults’ well-being through the intermediation role of place attachment.

### 2.4 Theoretical framework

**Figure 1:**
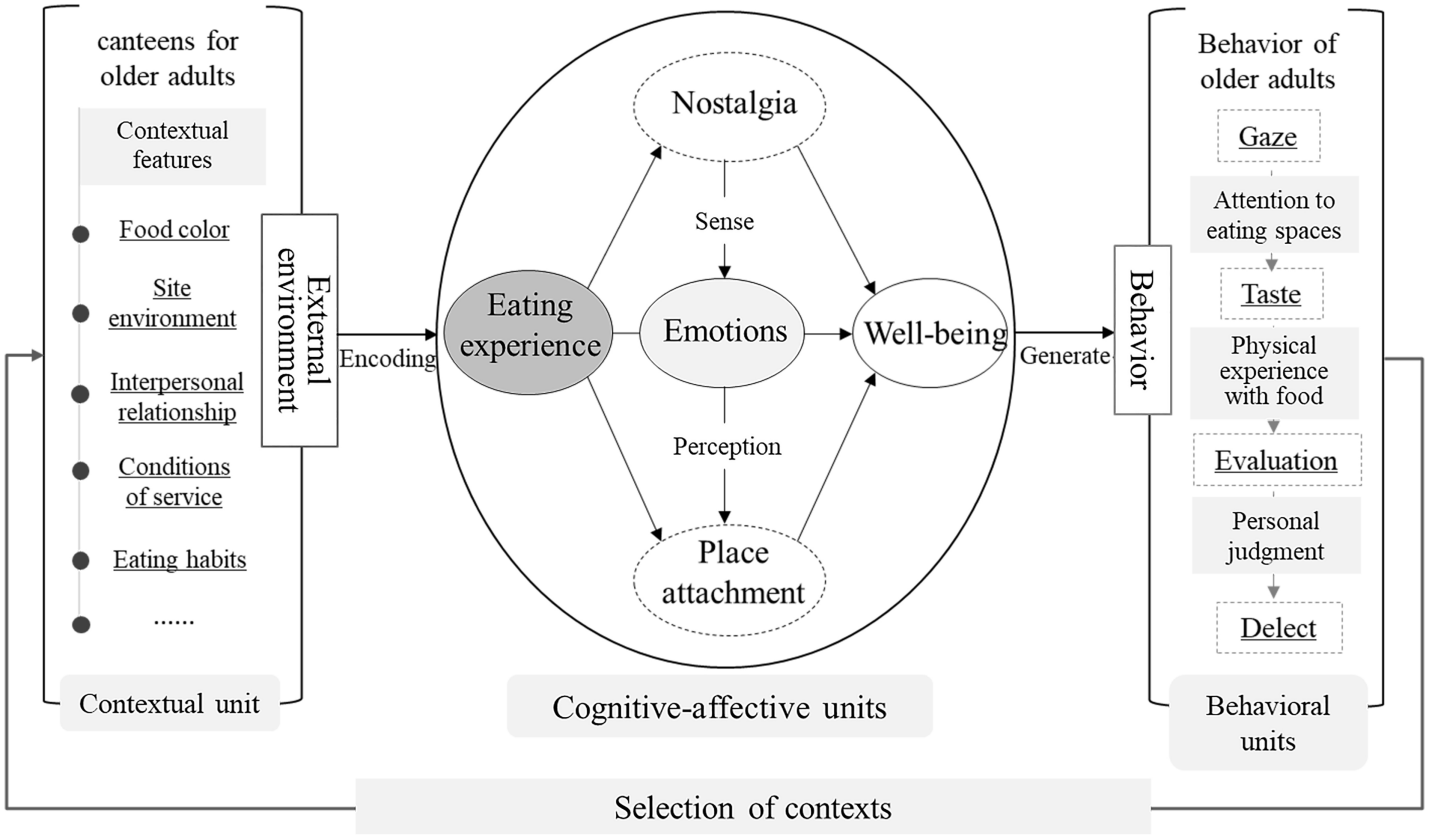
Theoretical framework of cognitive-affective personality systems.

Older adults’ well-being is a psychological activity in which individuals subjectively perceive food in a specific space, which includes older adults’ cognitive experience of food, emotional transfer, and psychological evaluation process (73). Therefore, based on the theory of human-environment relations and the cognitive-emotional personality systems theory, the study synthesizes a theoretical framework for exploring the relationship between eating experience, nostalgia, place attachment, and the well-being of older adults (Figure 1). Based on the framework and in combination with the hypotheses proposed in the previous section, a structural model for the study of the relationship between the eating experience of the older adults and their sense of well-being in the context of the community canteens for older people is constructed. Cognitive-affective personality systems (CAPS) theory has been used to explain the commonalities and differences in cognitive, affective, and behavioral responses of individuals in the same environment, as well as the influence of the environment on individual cognition and behavior (74). Characteristics of the external environment in which an individual lives can activate cognitive-affective units (CAUs) in the personality system and have an impact on the individual’s behavior. Cognitive-affective units in CAPS theory refer to all of an individual’s mental representations, which are mainly composed of elements such as encoding, expectancies and beliefs, affect, goals and values, competence, and self-regulated polarization (75).

In the external environment with the community canteens for older people as the context, the contextual features consisting of food and space are an important source of coding in CAPS; the affective element involves the older adults’ eating sensations, moods, and emotional responses (including physiological responses), which are differentiated through the older adults’ perceptual processes in the contextual unit of the dining canteens for the older adults into the nostalgia of the food and the place attachment to the space and well-being. Eating experience replaces the “coding” element in CAPS, which is closely related to other elements in CAUs and belongs to the cognitive part of the situational characteristics. Emotion is not only the result of cognition of situational characteristics but also a factor that influences individual cognitive and behavioral differences. CAUs in the process of interaction with the external environment, the different elements in the personality network system will be activated by certain situation characteristics, but also by other elements in the system, and ultimately generate a series of epiphenomenal behaviors, such as the older adults in the community canteens may exist in the gaze, tasting, evaluation, and selection behavior. Behaviors generated by the CAPS not only affect the external environment but also influence individual older adults’ choice of situations.

## 3 Materials and methods

### 3.1 Measurement methods

The study measured older adults’ eating experience, nostalgia, place attachment, and well-being with multi-structured questionnaire item scales, which were borrowed and adapted from representative studies by related scholars. The older adult Eating Experience Measure includes three questions: “Eating in the community canteens makes me feel happy,” “The food in the community canteens is nutritious and reassuring,” and “The food reduces my body fatigue in the community canteens.” These questions were modified from the Food Involvement Scale proposed by Bell et al. (76) and the Food Obsession Scale proposed by Gearhardt et al. (77), providing a reference for our research. Nostalgia involves a total of five questions, mainly referring to the Nostalgia Scale proposed by Holak and Havlena (78), Routledge et al. (32), and others. The six questions of the Place Attachment Scale were modified mainly concerning the Place Attachment Scale proposed by Williams and Vaske (79), Vada et al. (80), and others. Referring to the studies on the Well-being Scale by Ryan and Deci (9), Diener et al. (33), etc., four questions were identified to measure the well-being of the older adults, each with practical implications for reflecting their cognitive and emotional changes. These include “I am satisfied with my current state of life,” “My physical and spiritual needs are greatly satisfied,” “I have a new understanding of my true self through food,” and “I can feel the meaning and fulfilment of life after eating.” The study used a Likert 7-point scale to rate the eighteen questions from 1 = strongly disagree to 7 = strongly agree. The first part of the questionnaire dealt with eating experiences, nostalgia, place attachment, and well-being measurement items for older adults. The second part of the questionnaire contained demographic characteristics of the research participants and information on the eating behaviors of older adults, such as gender, age, family relationship, education level, and consumption expenditures.

### 3.2 Data collection and sample characteristics

In the study of eating experience, nostalgia, place attachment, and well-being of older adults, we chose to use the Guangzhou community canteens as a space context to investigate the eating experience and well-being of the older adults who dine in the community canteens. Guangzhou, a vital mega-city in China, is the center of exchange and fusion of Guangfu, Hakka, and Chaoshan cultures. From the point of view of the inheritance and evolution of Lingnan dietary culture, the unique geographic location and climatic conditions have made Guangzhou’s dietary culture present the qualities of diverse ingredients, light flavors, fine cooking, and delicious dishes. There has been a saying in China since ancient times that “eating in Guangzhou” (81). Guangzhou’s regional food, Cantonese cuisine, has long been an identity marker in the hearts of many people. Specialties such as morning tea, barbecued pork, and intestinal noodles carry the place identity and emotional attachment of residents to Lingnan culture. However, as globalization continues to accelerate, the globalization of dietary culture has impacted Canton culture to varying degrees. It has affected Guangzhou resident’s perceptions, attitudes, and actions toward regional food culture (82). At the same time, the population aging trend in Guangzhou has intensified, with 1.42 million older adults aged 65 and over accounting for 13.75 percent of the household population. Among the older adult population, the proportion of empty nesters, singletons, and migrants is significant, and community service facilities for older people face multiple pressures. Although the Guangzhou Municipal Government has established 1,088 community canteens as a means of upgrading the level of provision of service facilities and building a community service system for older people, making older adults eat more happily is still a significant challenge for Guangzhou in promoting its active aging strategy.

In this context, based on the Chinese Healthy Older Adults standard issued by the National Health Commission of the People’s Republic of China (NHCPRC), the target population of the survey was limited to older adults aged 65 years and above who are living on their own or basically on their own. Data collection was divided into three main stages. First, based on the spatial layout and population matching of the community canteens in Guangzhou, 10 canteens in each district were selected as the sample data collection sites from 1,088 community canteens in 176 streets (towns) in 11 districts, including Yuexiu, Haizhu, and Tianhe. Secondly, for the 110 canteens selected, the eligibility criteria for participating in the survey were set based on the criterion of “healthy older adults with more than 10 dining experiences per month.” Third, to improve the credibility of the questionnaire data and to reflect the participants’ real personal emotional will, 158 samples were initially collected through the pre-survey to test the questions. The formal survey and data collection were randomly distributed in the form of questionnaires, and the data collection process was terminated after 63 days (January 10 to March 13, 2024), with a total of 643 samples of data collected, of which 128 were invalid samples of data, and a total of 515 samples of data were used in the hypothesis testing process (see from Table 1).

**Table 1:** Basic characteristics of the sample.

Demographic characteristics		Sample size/person	Ratio/%
Genders	Male	240	46.60
	Women	275	53.40
Age	60–65 years	197	38.25
	65–70 years	156	30.29
	70–75 years	67	13.01
	75–80 years	85	16.50
	80 years and over	10	1.94
Family relations	Childless	9	1.75
	An only child	247	47.96
	Many children	259	50.29
Educational attainment	Primary school	38	7.38
	Junior high school	176	34.17
	Senior high school	190	36.89
	Bachelor’s degree or above	111	21.56
Catering consumption	Less than 500 CNY	41	7.96
	500–800 CNY	31	6.02
	800–1,000 CNY	174	33.79
	1,000–1,200 CNY	123	23.88
	1,200–1,500 CNY	47	9.13
	1,500–2000 CNY	64	12.43
	More than 2000 CNY	35	6.80

### 3.3 Data analysis

Before the measurement model analysis, the multivariate normal distribution of all sample data was examined through skewness and kurtosis analysis, with the skewness index ranging from −1.314 to −0.563 and the kurtosis index ranging from −0.727 to 1.280, which meets the requirement of the absolute value of the skewness index being less than 3, and the absolute value of the kurtosis index being less than 8 (83), which indicates that the research sample data presents a normal distribution, which meets the assumption of normality. The study chooses the PLS-SEM method to test the measurement of the model. PLS-SEM is suitable for the analysis of constitutive models, complex models, and higher-order models, and it has a typical advantage in the analysis of multiple mediation models (84). On this basis, SmartPLS 4.1.0 software was selected for evaluating the measurement and structural models. Since the study data were obtained through a questionnaire-item survey, common method variance (CMV) was assessed based on Harman’s one-way test and variance inflation factor (VIF) before PLS-SEM analysis. The results of the exploratory factor analysis showed that the total variance explained by the single factor was 37.259%, which was below the threshold of 50% of the acceptable range of common method variance. In addition, the VIF values of the structural model ranged from 1.442 to 3.586, which are lower than the general value of 5, indicating that there is no problem of multicollinearity among the variables (85). The analysis of model results was divided into two parts: measurement model and structural model testing, and the significance test were performed on 515 cases and 5,000 subsamples with the help of the PLS-SEM Algorithm and Bootstrapping analysis.

## 4 Analysis of results

### 4.1 Measurement models

The key parameters of the measurement model include convergent validity, discriminant validity, and reliability (see from Table 2). From the data in the table, it can be seen that the Cronbach’s *a* and composite reliability (CR) values of all variables exceeded the standardized value of 0.7, which indicates that there is good internal consistency among the variables. Meanwhile, the standardized factor loadings and average variance extracted (AVE) values of all variables are greater than the standard value of 0.5, indicating good convergent validity among the variables (84). On this basis, to effectively address the shortcomings of PLS-SEM in overestimating factor loadings, the Fornell-Larcker criterion and heterotrait-monotrait ratio (HTMT) need to be utilized to measure the discriminant validity among different variables (see from Table 3). From the table, it can be seen that the square root of AVE for all factors is higher than the correlation between a factor and all other factors in the model, and the correlation value of each factor is lower than the threshold value of 0.85 for the HTMT ratio (86), which indicates that the model’s discriminant validity is significant.

**Table 2:** Measurement model test.

Variant	Items	Mean	Loadings	α	CR	AVE
Eating experience	Eating in the community canteens makes me feel happy (EE1)	4.89	0.708	0.744	0.838	0.636
	Nutritional matching of food in the community canteens (EE2)	4.84	0.757			
	My physical fatigue is reduced through the food in the community canteens (EE7)	4.69	0.914			
Nostalgia	I remember some happy experiences with this place (NOS1)	5.23	0.806	0.907	0.931	0.729
	A particularly important deceased person/friend comes to mind here (NOS2)	5.18	0.862			
	I recall scenes from my life growing up (NOS4)	4.99	0.907			
	I miss the taste of the food I used to eat in the cafeteria when I was a kid (NOS 5)	5.04	0.851			
	I am reminded of an extremely memorable time (NOS7)	5.03	0.840			
Attachment place	Feeling different when eating at the community canteens than when eating the same food at home (AP5)	5.12	0.811	0.887	0.914	0.641
	The community canteens are more important than other restaurants (AP6)	5.09	0.829			
	I feel comfortable when I am in an community canteen environment (AP7)	5.49	0.774			
	The community canteens have unique significance and value to my daily life (AP8)	5.29	0.855			
	The community canteens have a sense of communal belonging for me (AP9)	5.21	0.806			
	The ambiance of the community canteens gives me a feeling of home (AP10)	5.31	0.721			
Older adults’ well-being	I am satisfied with my current state of life (OWB1)	4.92	0.810	0.874	0.914	0.726
	My physical and spiritual needs are greatly satisfied (OWB2)	5.05	0.829			
	Through food, I gained a new understanding of myself (OWB4)	4.91	0.888			
	Eating can give me a sense of meaning in life (OWB6)	5.03	0.880			

**Table 3:** Fornell-Larker criteria and HTMT ratios.

Variant	Place attachment	Eating experience	Older adults’ well-being	Nostalgia
Place attachment	**0.800**			
Eating experience	0.107 (0.124)	**0.798**		
Older adults’ well-being	0.656 (0.729)	0.103 (0.117)	**0.852**	
Nostalgia	0.419 (0.464)	0.351 (0.377)	0.245 (0.267)	**0.854**

Bold diagonal lines indicate the square root of the variable AVE.

### 4.2 Structural models

The test of the structural model involves key metrics such as model fitness (SRMR value), coefficient of determination (*R^2^*), redundancy index for blindfolded cross-validation (*Q^2^*), and variance inflation factor (VIF) (84). The study examined the path coefficients, t-values, and VIF values among different variables in the model based on Bootstrapping analysis of PLS-SEM (Table 4). The SRMR value in the structural model is 0.067, which is lower than the standard threshold of 0.08, indicating that the model is adequately fitted (86). Meanwhile, the *Q^2^* values of the structural model were all greater than 0, the internal VIF values were less than the threshold value of 3, and the *R^2^* values of all latent variables exceeded the threshold value of 0.2 level, which indicated that the structural model had good validity and fit validity.

**Table 4:** Structural model tests and path coefficients.

Hypothesis	Pathway relationship	*β*	*t*	*p*	VIF	Result
H1	Eating experience → Nostalgia	0.351***	8.952	0.000	1.000	Favor
H2	Eating experience → Place attachment	0.107*	2.289	0.022	1.000	Favor
H3	Eating experience → Older adults’ well-being	0.103*	2.244	0.025	1.143	Favor
H4	Nostalgia → Older adults’ well-being	−0.055	1.326	0.185	1.371	Rejection
H5	Place attachment →Older adults’ well-being	0.673***	21.633	0.000	1.216	Favor
H6	Eating experience → Nostalgia →Older adults’ well-being	−0.019	1.272	0.203		Rejection
H7	Eating experience → Place attachment →Older adults’ well-being	0.072*	2.285	0.022		Favor

***p < 0.001; **p < 0.01; *p < 0.05; Nostalgia R^2^ = 0.301, Q^2^ = 0.176; Place attachment R^2^ = 0.223, Q^2^ = 0.117; Older adults’ well-being R^2^ = 0.433, Q^2^ = 0.285.

From the results in the table, hypotheses H1, H2, H3, H5, and H7 were supported, and hypotheses H4 and H6 were rejected. H1 verified the effect of older adults’ eating experience in the community canteens on their nostalgia. The results showed a positive and significant effect of older adults’ eating experience in the community canteens on the production of their nostalgia (*β* = 0.351, *p* < 0.001). This hypothesis is consistent with the findings of established studies (6, 24, 63), suggesting that specific types of food have the function of storing past experiences of the consumer; when behaviors related to the consumption and experience of food take place in a similar space, individual memories coalesced in the food will be awakened, manifested as positive or negative emotions about the individual’s past experiences. H2 verified the effect of older adults’ eating experience in the community canteens on their place attachment. The results showed that older adults’ eating experience had a positive effect on their place attachment (*β* = 0.107, *p* < 0.05). Older adults construct a view of place based on their individual cognitive-emotional and cultural value systems and generate a place identity and emotional attachment regarding food through their physical and emotional experience of food in the community canteens (40, 41). H3 reflects that the positive effect of eating experience on older adults’ well-being (*β* = 0.103, *p* < 0.05) is fully established, eating experience not only satisfies their basic needs but also enhances their pleasure through the perceived emotional value attached to the food.

H4 and H5 verified the effects of nostalgia and place attachment on older adults’ well-being. The results showed that nostalgia generated by older adults in the community canteens did not have a positive effect on the formation of well-being (*β* = −0.055, *p* = 0.185), suggesting that positive or negative nostalgia does not make older adults feel happier. Nostalgia, even though it can increase the sense of meaning in life and alleviate loneliness among older adults (63), nostalgia generated in the community canteens is a possible impediment to older adults’ well-being. For example, the community canteens stimulates negative emotions about the past famine experience, which in turn weakens older adults’ judgment of happiness in the present; it does not bring about an increase in happiness due to the contrasting differences in material living conditions between the past and the present. H5 reflects that the positive effect of place attachment on older adults’ well-being (*β* = 0.673, *p* < 0.001) is extremely significant, indicating that older adults develop a strong life dependence and place belonging to the food and environment in the community canteens and that the canteens sustain the generation of well-being while meeting the daily physiological needs of the older adults, which is consistent with the findings of the established studies (66).

To investigate how eating experience indirectly affects the generation of well-being in older adults through the intermediation role of nostalgia and place attachment, the study conducted an intermediation analysis in SmartPLS 4.1.0. The test results showed that nostalgia did not have a significant mediating moderating effect on eating experience and well-being (*β* = −0.019, *p* = 0.203), indicating that nostalgia gained by older adults in the community canteens could not indirectly modulate the relationship between older adults’ eating experience and well-being. Place attachment had a significant mediating moderating effect on the relationship between eating experience and well-being (*β* = 0.072, *p* < 0.05), suggesting that older adults’ place attachment to the canteens is an important factor influencing the relationship between eating experience and older adults’ well-being. It also suggests that the reason why older adults eat more happily is not influenced by the positive or negative emotions embedded in the eating environments but is attributed to the sense of identity and attachment to food and place that older adults develop during the eating experience.

## 5 Discussion

### 5.1 Theoretical contributions

From the theoretical tendency of well-being research, two different forms of well-being have been mainly formed: hedonic and realized well-being (87). Hedonic well-being focuses on the subjective pleasure of the person and emphasizes the pleasure of having needs met without any hard work, while realized well-being emphasizes that well-being arises from the process of seeking the self and realizing the self (9). Previous research has distinguished between subjective and psychological well-being (9, 52). However, some views have suggested that subjective well-being and psychological well-being have some conceptually related characteristics (88) and that the essence of well-being should be a unified combination of subjectivity and objectivity, pleasure and meaning, and enjoyment and development. Based on the above viewpoints, the study defines older adults’ well-being as “the fulfillment of self-needs and the realization of life meaning in the process of pursuing the psychological experience of well-being in the older adults,” to bridge the research differences caused by the subjective and objective differences in the connotation of well-being.

The study is based on the theory of human-environment relations, and cognitive-emotional personality systems (CAPS) theory and synthesizes and proposes a theoretical framework for exploring the relationship between eating experience, nostalgia, and place attachment and older adults’ well-being in the community canteens. Older adults’ well-being is older adults’ evaluation of the state and quality of their own lives (33), and older adults’ well-being under the influence of eating experience includes both reflective cognitive judgments about food, such as life satisfaction, and emotional reactions to food in the external environment. Nostalgia and place attachment triggered by food are important sources of well-being in older adults (24), which reflects the physical experience and emotional cognitive process of older adults in the interaction between food and the external environment and is a product of the interaction between human-environment relationships. Older adults’ subjective perception of food in the process of pursuing the meaning of life reflects that people have a special emotional orientation to the external environment and the things in the environment. CAPS theory emphasizes that the characteristics of the external environment in which an older individual lives can activate the cognitive-emotional units (CAUs) in the personality system and have an impact on the choice of individual behavior, which to some extent compensates for the self-determination theory’s understanding of individual self-motivation realization of the external environmental triggers excluded.

In the context of the rapid development of food commoditization, people feel nostalgic for food cultures, and specific foods become tools for reconnecting with the lost past. Currently, the experience of food is linked to sensory memory, historical identity, national identity, and nostalgia (5, 6, 8), and most studies emphasize the exploration of food symbolism. However, the integration of nostalgia and place attachment as effective ways to enhance individual well-being with the experience of eating and the well-being of older adults has received less attention (32, 89). Therefore, exploring the pleasure of eating and the intrinsic associations between eating experiences and well-being in older adults provides new insights into the interplay between food consumption, emotional experiences, and individual memories.

### 5.2 Practical implications

From 1997, when the concept of “active aging” was put forward by the international community, to 2023, when China’s institutional framework for actively responding to population aging will have been initially established, the policy framework for active aging, which revolves around the three pillars of health, security, and participation of older adults, is gradually being improved. However, the existing policies mainly focus on the construction of service for older people, ignoring the multi-directional integration of policies with the daily lives of older adults, such as the integration of policies with food, industry, science and technology, education, etc. Given this, clarifying the role of the relationship between the eating experience of older adults and well-being in the community canteens is the key to measuring the implementation of the policy on the ground. The results of the study suggest that older people’s eating experience further affects the generation of older adults’ well-being through the role of place attachment, whereas older adults’ nostalgia in the community canteens does not stimulate their nostalgia. Therefore, the study will provide an empirical basis for governmental departments to formulate action plans for the construction of the community canteens, suggesting measures to enhance the locality of the canteens through the construction of local specialty recipes and the rational layout of the facility space; at the same time, it will enhance older adults’ place attachment to the community canteens as a means of enhancing well-being. Specifically, government departments and community service providers should make corresponding improvements at the levels of physical health, food consumption, and dietary environment of older people.

First, dietary management files must be established based on the dietary habits, physical conditions, and behavioral characteristics of older people in different age groups. By judging the physical health of older people, standards for older adults’ service will be formulated on a graded and categorized basis; at the same time, recreational activities for older people will be actively carried out to prevent them from falling into a state of anxiety, depression, and loneliness.

Secondly, organic green food is chosen as the main ingredient, and local Chinese medicine culture is integrated to build a unique recipe for a healthy diet for older people. On the one hand, it is necessary to emphasize the regulation of food prices and improve the financial security mechanism for the community canteens; on the other hand, food safety is a matter of health for older people, and it is necessary to strengthen the inspection of food hygiene.

Lastly, older people are susceptible to the perception of the dietary environment, which must be age-adapted in light of older adults’ memories. Based on focusing on environmental health and safety, the colors of the dietary environment should be warm and straightforward, and the architectural and interior design must be in line with the living habits and ergonomic characteristics of older people.

### 5.3 Research limitations

The study also has certain limitations, specifically: first, it is limited by the less attention paid by established studies to the relationship between eating experience, nostalgia, place attachment, and the well-being of older adults, as well as the individual variability of older adults’ eating habits, food preferences, and physical experience. The study did not differentiate the measurement dimensions of different variables when sorting out the variable relationships but integrated the dimensions of different variables in a questionnaire scale for unified measurement, which had some impact on the complexity of the model. Therefore, in future research, the intrinsic relationship between different dimensions of eating experience, nostalgia, place attachment, and well-being of older adults will be further explored. Second, in terms of the selection of research cases and objects, the study case of Guangzhou, a mega-city, was used to explore the relationship between the eating experience and well-being of older adults in the community canteens, and the generalizability of its findings was limited; at the same time, the research objects were limited to healthy older people aged 65 years old and above, which ignores the impact of the retirement age threshold (50/55 years old) of female workers in China’s retirement policy. Therefore, future research will expand the scale range of the study cases to explore the well-being of older adults in multiple age groups at the small and medium-sized city scale. Third, the current research model only considers the direct and mediating effects among different variables and pays insufficient attention to the moderating effects. Future research will expand the moderating variables such as gender differences, education background, and economic level, and further explore the relationship between variables such as sense of meaning in life and pro-social behaviors with eating experience and well-being of older adults, to better predict the affective changes and behavioral directions of older adults’ well-being.

## 6 Conclusion

The study takes the Guangzhou community canteens as a specific context to investigate the role relationship between eating experience, nostalgia, place attachment, and older adults’ well-being based on the CAPS. The main conclusions are as follows:

Older adults’ eating experience in the spatial context of the community canteens has a significant effect on the generation of well-being. As older adults are at the end of their life cycle, the abnormal changes in their body functions stimulate their special physiological needs for food; at the same time, the recollection of their past life experiences awakens their emotional memories of their life scenes. The physical and emotional needs for food derived from the spatial context of the community canteens are transformed into a physical action for food in the CAUs structure of older adults. The study further confirms that the process of “eating” in the community canteens can make older adults happier.

While exploring the relationship between older adults’ eating experience and well-being, the study found that older adults would give emotional evaluations of the food and space in the community canteens for older adults based on the accumulated differences in self-perceptions and form nostalgic emotions and place attachment specific to the canteens. It also shows that the canteens satisfy the physical needs of older adults as well as provide them with a strong emotional attachment and support. The results of the study confirm that there is a significant positive correlation between eating experience and nostalgia and place attachment and that the eating experience is more likely to stimulate older adults’ nostalgia.

It was found that nostalgia among older adults in the community canteens does not have a direct effect or will hurt the generation’s well-being. At the same time, older adults’ place attachment in the community canteens will significantly enhance their well-being. This finding also suggests that older adults are more concerned with the sense of security and belonging provided by the canteens, which can be inferred from the fact that older adults’ emotional perception is lower than their physical perception. In addition, the study also found that older adults’ place attachment was a bridge between eating experience and well-being and that eating experience did not affect well-being through nostalgia.

## Data Availability

The raw data supporting the conclusions of this article will be made available by the authors, without undue reservation.

## References

[1] Holtzman JD. In a cup of tea: commodities and history among Samburu pastoralists in northern Kenya. Am Ethnol. (2003) 30:136–55. doi: 10.1525/ae.2003.30.1.136

[2] Zhao R. History of Chinese food culture. Shanghai: Shanghai People's Publishing House, (2006). p. 329–334.

[3] Peng Z. Food anthropology. Beijing: Peking University Press, (2013). p. 118–125.

[4] Xu H. A history of Chinese cuisine: Six volumes. Hangzhou: Hangzhou Publishing House, (2014). p. 432–445.

[5] Sutton D. Whole foods: revitalization through everyday synesthetic experience. Anthropol Hum. (2000) 25:120–30. doi: 10.1525/ahu.2000.25.2.120

[6] Holtzman J. Food and memory. Annu Rev Anthropol. (2008) 35:361–78. doi: 10.1146/annurev.anthro.35.081705.123220

[7] Cheung S Wu DYH. The globalizations of Chinese food. 1st Ed. London, UK: Routledge, (2002). p. 61–85.

[8] Lupton D. Food, the body and the self. 1st Ed. London, UK: SAGE Publications, Ltd (1996). p. 75–94.

[9] Ryan R Deci E. On happiness and human potentials: a review of research on hedonic and eudaimonic well-being. Annu Rev Psychol. (2001) 52:141–66. doi: 10.1146/annurev.psych.52.1.141 11148302

[10] Lee JS Johnson MA Brown A Nord M. Food security of older adults requesting older Americans act nutrition program in Georgia can be validly measured using a short form of the U.S. household food security survey module. J Nutr. (2011) 141:1362–8. doi: 10.3945/jn.111.139378, PMID: 21562242

[11] Solbak NM Xu J-Y Vena JE Csizmadi I Whelan HK Robson PJ. Diet quality is associated with reduced incidence of cancer and self-reported chronic disease: observations from Alberta's tomorrow project. Prev Med. (2017) 101:178–87. doi: 10.1016/j.ypmed.2017.06.009 28601618

[12] Browning CJ Qiu Z Yang H Zhang T Thomas SA. Food, eating, and happy aging: the perceptions of older Chinese people. Front Public Health. (2019) 7:73. doi: 10.3389/fpubh.2019.00073 31024875 PMC6460243

[13] Luo L Han P. Assessing food-evoked emotions using functional magnetic resonance imaging: a systematic review. Food Qual Prefer. (2023) 108:104877. doi: 10.1016/j.foodqual.2023.104877

[14] IshikawaMYokoyamaTHayashiFTakemiYNakayaTFukudaY. Subjective well-being is associated with food behavior and demographic factors in chronically ill older Japanese people living alone. J Nutr Health Aging. (2018) 22:341–53. doi: 10.1007/s12603-017-0930-3, PMID: 29484347

[15] Lakoff G Johnson M. The metaphorical structure of the human conceptual system. Cogn Sci. (1980) 4:195–208. doi: 10.1207/s15516709cog0402_4

[16] Scott ML Vallen B. Expanding the lens of food well-being: an examination of contemporary marketing, policy, and practice with an eye on the future. J Public Policy Mark. (2019) 38:127–35. doi: 10.1177/0743915619831647

[17] Gallese V Sinigaglia C. The bodily self as power for action. Neuropsychologia. (2010) 48:746–55. doi: 10.1016/j.neuropsychologia.2009.09.038 19835895

[18] Riva G. The neuroscience of body memory: from the self through the space to the others. Cortex. (2018) 104:241–60. doi: 10.1016/j.cortex.2017.07.013, PMID: 28826604

[19] Brady J. Cooking as inquiry: a method to stir up prevailing ways of knowing food, body, and identity. Int J Qual Methods. (2011) 10:321–34. doi: 10.1177/160940691101000402

[20] Conde-Caballero D Rivero-Jimenez B Mariano-Juarez L. Memories of hunger, continuities, and food choices: an ethnography of the elderly in Extremadura (Spain). Appetite. (2021) 164:105267. doi: 10.1016/j.appet.2021.105267, PMID: 33933550

[21] Leichter D. Collective identity and collective memory in the philosophy of Paul Ricoeur. Études Ricoeuriennes/Ricoeur Studies. (2012) 3:114–31. doi: 10.5195/errs.2012.125

[22] Kim S Ellis A. Noodle production and consumption: from agriculture to food tourism in Japan. Tour Geogr. (2015) 17:151–67. doi: 10.1080/14616688.2014.978812

[23] Barrett FS Grimm KJ Robins RW Wildschut T Sedikides C Janata P. Music-evoked nostalgia: affect, memory, and personality. Emotion. (2010) 10:390–403. doi: 10.1037/a0019006, PMID: 20515227

[24] Hepper EG Ritchie TD Sedikides C Wildschut T. Odyssey's end: lay conceptions of nostalgia reflect its original Homeric meaning. Emotion. (2012) 12:102–19. doi: 10.1037/a0025167, PMID: 21859192

[25] Polletta F Callahan J. Deep stories, nostalgia narratives, and fake news: storytelling in the trump era. Am J Cult Sociol. (2017) 5:392–408. doi: 10.1057/s41290-017-0037-7

[26] Li B Zhu Q Li A Cui R. Can good memories of the past instill happiness? Nostalgia improves subjective well-being by increasing gratitude. J Happiness Stud. (2023) 24:699–715. doi: 10.1007/s10902-022-00616-0 36644477 PMC9826762

[27] Davis F. Yearning for yesterday: A sociology of nostalgia. New York: Free Press, (1979). p. 184–191.

[28] Sedikides C Wildschut T Baden D. Nostalgia: conceptual issues and existential functions In: Handbook of experimental existential psychology. New York, NY, US: The Guilford Press (2004). 200–14.

[29] Wildschut T Sedikides C Arndt J Routledge C. Nostalgia: content, triggers, functions. J Pers Soc Psychol. (2006) 91:975–93. doi: 10.1037/0022-3514.91.5.975 17059314

[30] Gammon S Ramshaw G. Distancing from the present: nostalgia and leisure in lockdown. Leis Sci. (2021) 43:131–7. doi: 10.1080/01490400.2020.1773993

[31] Leunissen J Wildschut T Sedikides C Routledge C. The hedonic character of nostalgia: an integrative data analysis. Emot Rev. (2021) 13:139–56. doi: 10.1177/1754073920950455

[32] Routledge C Wildschut T Sedikides C Juhl J. Nostalgia as a resource for psychological health and well-being. Soc Personal Psychol Compass. (2013) 7:808–18. doi: 10.1111/spc3.12070

[33] Diener E Oishi S Tay L. Advances in subjective well-being research. Nat Hum Behav. (2018) 2:253–60. doi: 10.1038/s41562-018-0307-6 30936533

[34] Sutton DE. Food and the senses. Annu Rev Anthropol. (2010) 39:209–23. doi: 10.1146/annurev.anthro.012809.104957

[35] Reid C Green J Buchmaier S McSween D Wildschut T Sedikides C. Food-evoked nostalgia. Cognit Emot. (2022) 37:1–15. doi: 10.1080/02699931.2022.2142525 36331076

[36] Li X Kong W Yang F. Authentic food experiences bring us back to the past: an investigation of a local food night market. J Travel Tour Mark. (2021) 38:233–46. doi: 10.1080/10548408.2021.1902910

[37] Kolas A Thowsen MP. On the margins of Tibet: cultural survival on the Sino-Tibetan frontier. New York, US: University of Washington Press, (2011). p. 123–147.

[38] Vignolles A Pichon P-E. A taste of nostalgia. Qual Mark Res Int J. (2014) 17:225–38. doi: 10.1108/QMR-06-2012-0027

[39] Wang S Lehto X Cai L Behnke C Kirillova K. Travelers' psychological comfort with local food experiences and place attachment. J Hospital Tour Res. (2023) 47:1453–77. doi: 10.1177/10963480211058474

[40] Tsai C-T. Memorable tourist experiences and place attachment when consuming local food. Int J Tour Res. (2016) 18:536–48. doi: 10.1002/jtr.2070

[41] Hsu FC Scott N. Food experience, place attachment, destination image and the role of food-related personality traits. J Hosp Tour Manag. (2020) 44:79–87. doi: 10.1016/j.jhtm.2020.05.010

[42] Clark G Chabrel M. Measuring integrated rural tourism. Tour Geogr. (2007) 9:371–86. doi: 10.1080/14616680701647550

[43] Lee J Kyle G Scott D. The mediating effect of place attachment on the relationship between festival satisfaction and loyalty to the festival hosting destination. J Travel Res. (2012) 51:754–67. doi: 10.1177/0047287512437859

[44] Katarzyna JC. Modern Japanese cuisine: food, power and national identity. 1st Ed. London, UK: Reaktion Books Ltd, (2007). p. 89–103.

[45] Fischer J. Vegetarianism, meat and modernity in India. 1st Ed. Abingdon, UK: Routledge, (2023). p. 102–127.

[46] Casey ES. Remembering, second edition a phenomenological study. 2nd ed Indiana University Press (2000).

[47] Tuan Y-F. Space and place: humanistic perspective In: Gale S Olsson G, editors. Philosophy in geography. Dordrecht: Springer Netherlands (1979). 387–427.

[48] Hwang J Hyun SS. The impact of nostalgia triggers on emotional responses and revisit intentions in luxury restaurants: the moderating role of hiatus. Int J Hosp Manag. (2013) 33:250–62. doi: 10.1016/j.ijhm.2012.09.001

[49] Lewicka M. Place attachment: how far have we come in the last 40 years? J Environ Psychol. (2011) 31:207–30. doi: 10.1016/j.jenvp.2010.10.001

[50] Bimbi F. From unhealthy satiety to health-oriented eating: narratives of the Mediterranean diet, managing a chronic illness In: Segal MT Demos V, editors. Gender and food: From production to consumption and after. Bingley, UK: Emerald Group Publishing Limited (2016). 89–115.

[51] Mariani J F. How Italian food conquered the world. New York, US: Palgrave Macmillan, (2011). p. 135–152.

[52] Diener E Suh EM Lucas RE Smith HL. Subjective well-being: three decades of progress. Psychol Bull. (1999) 125:276–302. doi: 10.1037/0033-2909.125.2.276

[53] Strandberg TE Strandberg AY Pitkälä K Salomaa VV Tilvis RS Miettinen TA. Chocolate, well-being and health among elderly men. Eur J Clin Nutr. (2008) 62:247–53. doi: 10.1038/sj.ejcn.1602707 17327862

[54] Jaeger SR Vidal L Chheang SL Ares G. Consumer conceptualisations of food-related wellbeing: an exploration of wellbeing-related terms in four industrialised countries. Appetite. (2022) 179:106286. doi: 10.1016/j.appet.2022.106286, PMID: 36038074

[55] Schnettler B Miranda H Lobos G Orellana L Sepúlveda J Denegri M. Eating habits and subjective well-being: a typology of students in Chilean state universities. Appetite. (2015) 89:203–14. doi: 10.1016/j.appet.2015.02.008, PMID: 25675858

[56] Apaolaza V Hartmann P D'Souza C López CM. Eat organic-feel good? The relationship between organic food consumption, health concern and subjective wellbeing. Food Qual Prefer. (2018) 63:51–62. doi: 10.1016/j.foodqual.2017.07.011

[57] Vega-Zamora M Torres-Ruiz FJ Murgado-Armenteros EM Parras-Rosa M. Organic as a heuristic cue: what Spanish consumers mean by organic foods. Psychol Market. (2014) 31:349–59. doi: 10.1002/mar.20699

[58] Bublitz MG Peracchio LA Andreasen AR Kees J Kidwell B Miller EG. Promoting positive change: advancing the food well-being paradigm. J Bus Res. (2013) 66:1211–8. doi: 10.1016/j.jbusres.2012.08.014

[59] Goetzke B Nitzko S Spiller A. Consumption of organic and functional food. A matter of well-being and health? Appetite. (2014) 77:96–105. doi: 10.1016/j.appet.2014.02.012, PMID: 24630940

[60] Cornil Y Chandon P. Pleasure as a substitute for size: how multisensory imagery can make people happier with smaller food portions. J Mark Res. (2016) 53:847–64. doi: 10.1509/jmr.14.0299

[61] Liu S Li S Chen Y Zheng T. Examining relationships among food’s perceived value, well-being, and tourists’ loyalty. J Vacat Mark. (2023) 29:161–74. doi: 10.1177/13567667221080569

[62] Fedorikhin A Patrick VM. Positive mood and resistance to temptation: the interfering influence of elevated arousal. J Consum Res. (2010) 37:698–711. doi: 10.1086/655665

[63] Sedikides C Wildschut T. Nostalgia: a bittersweet emotion that confers psychological health benefits In: Wood AM Johnson J, editors. The Wiley handbook of positive clinical psychology. New York, US: Wiley Blackwell (2016). 125–36.

[64] Kelley NJ Davis WE Dang J Liu L Wildschut T Sedikides C. Nostalgia confers psychological wellbeing by increasing authenticity. J Exp Soc Psychol. (2022) 102:104379. doi: 10.1016/j.jesp.2022.104379

[65] Fang F Zhao Y Xi Z Han X Zhu Y. The impact of famine experience on middle-aged and elderly individuals’ food consumption: evidence from China. J Econ Ageing. (2023) 26:100472. doi: 10.1016/j.jeoa.2023.100472

[66] Ujang N Zakariya K. The notion of place, place meaning and identity in urban regeneration. Procedia Soc Behav Sci. (2015) 170:709–17. doi: 10.1016/j.sbspro.2015.01.073

[67] Aitken R Campelo A. The four Rs of place branding. J Mark Manag. (2011) 27:913–33. doi: 10.1080/0267257X.2011.560718

[68] Scannell L Gifford R. Place attachment enhances psychological need satisfaction. Environ Behav. (2017) 49:359–89. doi: 10.1177/0013916516637648

[69] Vada S Prentice C Hsiao A. The influence of tourism experience and well-being on place attachment. J Retail Consum Serv. (2019) 47:322–30. doi: 10.1016/j.jretconser.2018.12.007

[70] Pourfakhimi S Nadim Z Prayag G Mulcahy R. The influence of neophobia and enduring food involvement on travelers' perceptions of wellbeing—evidence from international visitors to Iran. Int J Tour Res. (2021) 23:178–91. doi: 10.1002/jtr.2391

[71] Pliner P. The effects of mere exposure on liking for edible substances. Appetite. (1982) 3:283–90. doi: 10.1016/S0195-6663(82)80026-3 7159080

[72] Sylvie AK Jiang Q Cohen N. Identification of environmental supports for healthy eating in older adults. J Nutr Gerontol Geriatr. (2013) 32:161–74. doi: 10.1080/21551197.2013.779621 23663214

[73] Hilgard ER. The trilogy of mind: cognition, affection, and conation. J Hist Behav Sci. (1980) 16:107–17. doi: 10.1002/1520-6696(198004)16:2<107::AID-JHBS2300160202>3.0.CO;2-Y, PMID: 11608381

[74] Mischel W Shoda Y. A cognitive-affective system theory of personality: reconceptualizing situations, dispositions, dynamics, and invariance in personality structure. Psychol Rev. (1995) 102:246–68. doi: 10.1037/0033-295X.102.2.246 7740090

[75] Mischel W. Personality coherence and dispositions in a cognitive-affective personality (caps) approach In: D. Cervone & Y. Shoda, editors. The coherence of personality: Social-cognitive bases of consistency, variability, and organization. New York, US: Guilford Press (1999). 37–60.

[76] Bell R Marshall DW. The construct of food involvement in behavioral research: scale development and validation. Appetite. (2003) 40:235–44. doi: 10.1016/S0195-6663(03)00009-6 12798781

[77] Gearhardt AN Corbin WR Brownell KD. Preliminary validation of the Yale food addiction scale. Appetite. (2009) 52:430–6. doi: 10.1016/j.appet.2008.12.003, PMID: 19121351

[78] Holak SL Havlena WJ. Feelings, fantasies, and memories: an examination of the emotional components of nostalgia. J Bus Res. (1998) 42:217–26. doi: 10.1016/S0148-2963(97)00119-7

[79] Williams D Vaske J. The measurement of place attachment: validity and generalizability of a psychometric approach. For Sci. (2003) 49:830–40. doi: 10.1093/forestscience/49.6.830

[80] Diener E. Subjective well-being. Psychol Bull. (1984) 95:542–75. doi: 10.1037/0033-2909.95.3.542 6399758

[81] Luo Q Ding S Pan K. Generational differences in the influence of exotic gastronomic culture on local residents' place identity in Guangzhou. Geogr Res. (2018) 37:1762–74.

[82] Yin D Yang R Lin J. Multiple perceptions and consumption practices of transnational restaurant in the context of globalization: a case study of Guangzhou Zagol Habesha Ethiopian restaurant. Tourism Tribune. (2023) 38:134–47.

[83] Kline RB. Principles and practice of structural equation modeling. 4th ed. New York, US: Guilford Press (2016). 534 p.

[84] Hair JF Risher JJ Sarstedt M Ringle CM. When to use and how to report the results of PLS-SEM. Eur Bus Rev. (2019) 31:2–24. doi: 10.1108/EBR-11-2018-0203

[85] Kock N. Common method bias in PLS-SEM: a full collinearity assessment approach. Int J e-Collaboration. (2015) 11:1–10. doi: 10.4018/ijec.2015100101

[86] Henseler J Ringle CM Sarstedt M. A new criterion for assessing discriminant validity in variance-based structural equation modeling. J Acad Mark Sci. (2015) 43:115–35. doi: 10.1007/s11747-014-0403-8

[87] Ryff CD Singer BH Palmersheim KA. Social inequalities in health and well-being: the role of relational and religious protective factors In: O. G. Brim, C. D. Ryff, & R. C. Kessler, editors. How healthy are we? A National Study of well-being at midlife. Chicago, US: The University of Chicago Press (2004). 90–123.

[88] Keyes CL Shmotkin D Ryff CD. Optimizing well-being: the empirical encounter of two traditions. J Pers Soc Psychol. (2002) 82:1007–22. doi: 10.1037/0022-3514.82.6.1007, PMID: 12051575

[89] Karagöz D Ramkissoon H. Nostalgic emotions, meaning in life, subjective well-being and revisit intentions. Tour Manag Perspect. (2023) 48:101159. doi: 10.1016/j.tmp.2023.101159

